# Effect of Nimodipine Treatment on the Clinical Course of Reversible Cerebral Vasoconstriction Syndrome

**DOI:** 10.3389/fneur.2019.00644

**Published:** 2019-06-18

**Authors:** Soohyun Cho, Mi Ji Lee, Chin-Sang Chung

**Affiliations:** ^1^Department of Neurology, Samsung Medical Center, Sungkyunkwan University School of Medicine, Seoul, South Korea; ^2^Neuroscience Center, Samsung Medical Center, Seoul, South Korea

**Keywords:** reversible cerebral vasoconstriction syndrome, nimodipine, thunderclap headache, clinical course, treatment

## Abstract

**Background:** In reversible cerebral vasoconstriction syndrome (RCVS), nimodipine is currently used for the treatment, although no evidence is available to support its disease-modifying effect. In this prospective observational study, we investigated whether earlier nimodipine treatment can modify the clinical course of reversible cerebral vasoconstriction syndrome.

**Methods:** We prospectively observed patients with angiogram-proven RCVS within 1 month after onset in the Samsung Medical Center between October 2015 and January 2018. Nimodipine was started in all patients immediately after diagnosis. Time from onset to the first nimodipine treatment was categorized as tertiles. We analyzed Kaplan-Meier curve and Cox proportional hazard model to test if the timing of nimodipine treatment can affect the clinical course of thunderclap headaches (TCHs) defined as the duration from onset to remission of thunderclap headaches.

**Results:** In 82 patients included in this study, 71 (86.6%) patients showed remission of TCHs after starting nimodipine treatment. When categorized into earliest (<6 days), early (6–13 days), and late (≥14 days) treatment groups, earlier treatment was significantly associated with shorter clinical courses (median, 2 days [interquartile range 1–3] vs. 7 days [4–10] vs. 10 days [5–15]; log-rank *p* < 0.001). Univariable and multivariable Cox regression analyses also demonstrated an independent effect of earlier nimodipine treatment on earlier remission of TCHs (adjusted hazard ratio, 0.75 per 1-day delay in treatment; 95% CI, 0.693–0.802, *p* < 0.001).

**Conclusions:** The clinical course of RCVS differed according to the timing of nimodipine treatment, suggesting the effect of earlier nimodipine treatment. In addition to preventing TCHs, beneficial effects of earlier nimodipine treatment on the progression of vasoconstriction and development of neurological complications should be investigated in future studies.

## Introduction

Reversible cerebral vasoconstriction syndrome (RCVS) is characterized by recurrent thunderclap headaches (TCHs) and multifocal vasoconstriction of the cerebral arteries and is fully reversible after 3–6 months (1). TCH is one of the most characteristic presentations of RCVS and often remains the only symptom (2–4). Although RCVS is considered self-limiting, a substantial proportion of patients can have neurological complications such as seizure, cerebral infarction, subarachnoid or intracerebral hemorrhage, and posterior reversible encephalopathy syndrome (PRES) during their disease course (2, 4–7). Nevertheless, no evidence-based treatment exists for RCVS because of the lack of animal models and randomized controlled trials.

Currently, nimodipine is most frequently used for the empirical treatment of RCVS. Many experts recommend the use of nimodipine based on clinical experience (1, 8–11). Among various calcium channel antagonists, nimodipine is characterized by its ability to cross the blood-brain barrier and its selective affinity to cerebral arteries (12). Nimodipine also exerts neurohormonal effects by inhibiting receptor agonists such as serotonin, catecholamines, and histamines and by inducing an increase in plasma adenosine levels (13–15). This can explain the role of nimodipine in preventing and restoring vasospasm, which is the main pathophysiology of RCVS. Thus, nimodipine can exert a beneficial effect on the treatment of RCVS.

In this observational study, we questioned whether nimodipine treatment can modify the disease course of RCVS. We tested if nimodipine treatment prevents the recurrence of TCHs and whether the timing (early vs. late) of treatment affects the clinical course (i.e., time from onset to remission of TCH) in RCVS.

## Methods

### Patients

We prospectively screened patients with TCHs who visited Samsung Medical Center, Seoul, South Korea from October 2015 to January 2018. Among the screened patients, those with angiogram-proven RCVS with a clearly remembered onset of <1 month were included in this study. Because a definite diagnosis of RCVS is often made after a certain period of observation, we included patients at their first presentation if they met the following criteria: patients who: (1) presented to us within 1 month after onset, (2) clearly remembered the date and mode of onset, and (3) did not have a secondary cause other than RCVS. After 3 months of observation, the diagnosis of RCVS was confirmed. We excluded patients who were finally classified into primary thunderclap headache or probable RCVS. All the diagnoses of RCVS and primary thunderclap headache were based on the third edition of the International Classification of Headache Disorders, beta version (ICHD-3 beta) (16). According to ICHD-3 beta, headache attributed to RCVS is manifested as typically recurrent TCHs, at least one TCH triggered by the typical precipitants and no recurrence of significant headache after 1 month. In contrast, TCHs which do not fit to the criteria for RCVS, probable RCVS, or other primary or secondary headache disorders are classified into primary thunderclap headache: e.g., single non-triggered TCH without any evidence of secondary causes or spontaneous TCHs recurring after 1 month after onset. In our hospital, nimodipine is routinely prescribed to all patients immediately when a diagnosis of RCVS is suspected. Some exceptional cases were excluded in this study. The Samsung Medical Center Institutional Review Board approved this study. Written consent was obtained for all patients at the inclusion visit.

### Clinical Evaluation

Our protocol for evaluation of TCH was described previously (17). To summarize, it depended on the site of recruitment: emergency room (ER), outpatient headache clinic, or inpatient consultation. From the ER, patients with acute onset severe headache were referred to a neurologist after aneurysmal subarachnoid hemorrhage (SAH) was excluded based on non-contrast brain CT and post-contrast CT angiogram findings. After referral, a combination of lumbar puncture, brain MRI, MR angiogram (MRA), and occasionally transfemoral cerebral angiography was performed for the differential diagnosis. The same protocol was applied in cases of inpatient consultation. In the outpatient headache clinic, patients were primarily evaluated using brain MRI and MRA, whereas patients with persistent headaches were referred to the ER and the emergency protocol was then applied.

All patients were interviewed by headache specialists (M.J.L. and C.S.C.). Patients completed a structured questionnaire on headache characteristics specifically designed for the evaluation of TCHs. We collected information on the onset of TCH, recurrence pattern (single or recurrent TCHs), the number of TCHs, severity, triggers for TCH (sexual activity, exertion, Valsalva maneuvers, emotion, bending, bathing and/or showering), and the presence of a comorbid migraine. After treatment, patients were serially followed-up by the same investigator to determine the recurrence of TCH and date of the last attack. All but one patient who underwent transcranial Doppler underwent MRA at the follow-up visit for the confirmation of reversibility.

In all patients, the degree of vasoconstriction was measured in all the first, second, and third branches of the intracranial arteries. Vasoconstriction was graded as 0, normal (normal flow signal); (1) <50% (focal indentation but >50% of lumen visualized); (2) 50–99% (>50% reduction of flow signal or flow gap but visible flow signals distal to the stenotic segment); and (3) occlusion (no distal flow signal visualized) (17, 18). Patients who showed grade ≥2 segmental vasoconstrictions in ≥2 intracranial arteries were included in this study. The extent of vasoconstriction was defined as the sum of affected segments with grade ≥2 vasoconstrictions in each patient. Any transient neurological symptoms reported by patients were recorded. We also assessed neurological complications such as seizure, ischemic stroke, cortical SAH, and PRES. BP surge was defined when a patient showed an intermittent rise in BP: systolic BP (SBP) of >160 mmHg during headache attacks or >30 mmHg higher than baseline [modified from Chen et al. (2)].

### Treatment

Oral nimodipine was prescribed immediately upon a diagnosis of suspected RCVS. Initial treatment was started at 30 mg every 8–12 h per day (median, 1.5 mg/kg/day). If TCHs effectively remitted with this regimen, the dose of nimodipine was unchanged for 3 months. In patients (*n* = 7, 8.5%) who had recurrent TCHs or persistent headache of more than moderate intensity, the dose of nimodipine was escalated and maintained when tolerated. We recorded the date of nimodipine administration and dose increment in all patients. We measured SBP and diastolic BP (DBP) at every visit.

### Treatment Effect on the Recurrence of TCH

**Figure 1:**
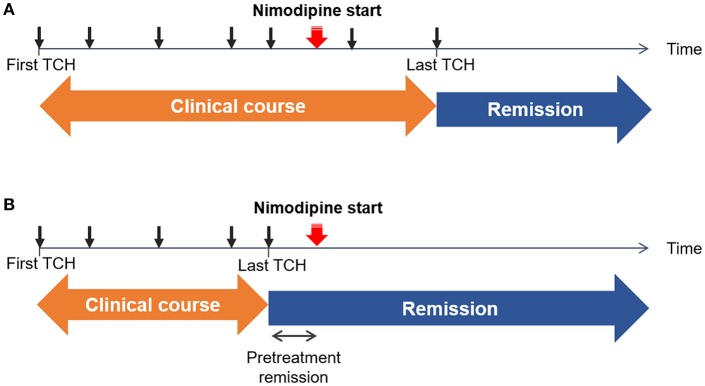
Schematic illustrations of example cases. The time of each thunderclap headache (TCH) is marked as a black arrow. The initiation of nimodipine treatment is marked with a red arrow. Remission was defined as the date of the last TCH. Clinical course was defined as a duration from the first TCH to the last TCH. **(A)** Non-response: TCHs did not remit immediately after the nimodipine administration **(B)** Response: TCHs remitted after the nimodipine administration. Since there can be an interval between the last TCH and nimodipine administration, we took the length of pretreatment remission period into consideration.

Patients were prospectively followed-up. All patients were scheduled to visit the hospital within 1 month after the initial treatment. Neuroimaging was followed-up after 3 months of treatment to confirm reversibility. If vasoconstrictions were not normalized, neuroimaging was followed-up at 6 months with treatment being continued. During follow-up visits, any recurrence of TCH was recorded, but a mild residual headache was not regarded as a recurrence. We recorded the dates of recurrence of TCH before and after nimodipine treatment. Remission of TCH was defined as no recurrence of TCH. The date of the last TCH before remission was identified to determine the clinical course, defined as the duration from onset to remission (i.e., the date of the last TCH). We also defined the pretreatment remission period as the interval from the last pre-treatment TCH to the nimodipine administration to consider the possibility that the patients were already in remission before the nimodipine treatment. The scheme of our definitions is illustrated in Figure 1.

### Statistical Analysis

Data are presented as the number (percentage) or median (interquartile range, IQR), or as otherwise specified. Categorical variables were compared with the chi-square test or Fisher's exact test, and continuous variables were analyzed using the Kruskal–Wallis test or Mann–Whitney U test. The Jonckheere–Terpstra test or linear-by-linear association analysis was used to assess trends among the groups. Time from onset to treatment was grouped into tertiles. Patient demographics and characteristics, neurological complications, extent of vasoconstriction, and remission rates were compared according to the tertiles of time from onset to treatment. To test if earlier administration of nimodipine affected the clinical course, we set the clinical course (duration from onset to remission of TCHs) as an outcome in the survival analysis. Survival curves were generated using the Kaplan–Meier method and compared among tertiles of time from onset to treatment with the log-rank test. In the univariable Cox proportional hazards model, timing of treatment was tested as a continuous variable. A multivariable Cox proportional hazards model was then used to test the independent effect of timing of treatment adjusted for age, sex, extent of vasoconstriction, length of pretreatment remission period, and neurological complications before treatment. For each Cox proportional hazards model, proportional hazards assumptions were examined by testing Schoenfeld residuals. The results are reported as hazard ratios (HR) with 95% CIs. All statistical analyses were performed using commercially available software (Stata 15.0; StataCorp LLC, College Station, TX, USA). A *p-*value of <0.05 was considered significant. Bonferroni correction was performed to correct for multiple comparisons among treatment groups.

## Results

### Patients

**Figure 2:**
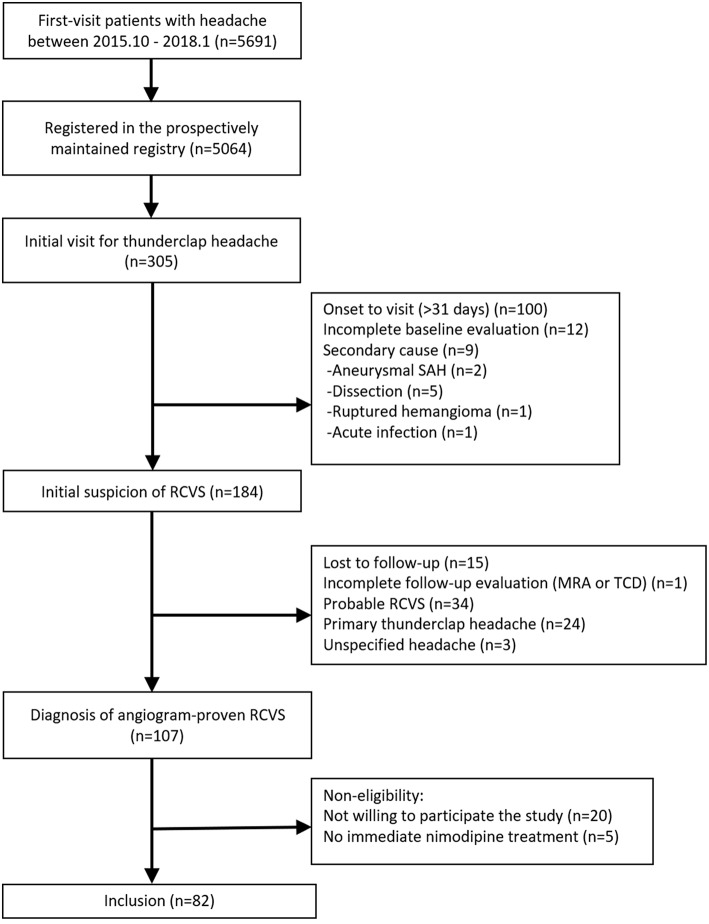
Flow diagram of the study.

The study flowchart is shown in Figure 2. We screened 305 patients with TCH during the study period. A total of 107 patients were diagnosed with angiogram-proven RCVS. After the exclusion of five patients who did not receive nimodipine treatment and 20 patients who were unwilling to participate in this study, 82 patients were finally included in this study. The baseline characteristics between included vs. excluded patients were not significantly different (Data not shown; available upon requests). The demographics and characteristics of included patients are summarized in Table 1.

**Table 1:** Demographics and characteristics of patients.

	**Patients (*n =* 82)**
Age (years)	52 (47–57)
Female sex	67 (81.7%)
Headache severity (NRS)	9 (8–10)
**Etiology**	
Postpartum	2 (2.4%)
Drug	2 (2.4%)
Idiopathic	78 (95.2%)
Comorbid migraine	12 (14.6%)
**Triggered by typical precipitants**	
At least one precipitant	64 (78.0%)
Multiple precipitants	30 (36.6%)
**Typical precipitants**	
Sexual activity	4 (6.3%)
Exertion	2 (3.1%)
Valsalva-like maneuver	35 (54.7%)
Emotion	17 (26.6%)
Bathing and/or showering	19 (29.7%)
Bending	18 (28.1%)
Focal neurological deficit	9 (11.0%)
**Neurological complication at the time of presentation**	
Any	7 (8.5%)
Seizure	1 (1.2%)
Ischemic stroke	1 (1.2%)
Cortical SAH	3 (3.7%)
Intracerebral hemorrhage	1 (1.2%)
PRES	1 (1.2%)
BP surge	31 (37.8%)
Extent of vasoconstriction	5 (3–7)

*Data are presented as median (IQR) or number (percent)*.

### Timing of Nimodipine Treatment

In the 82 included patients, 73 (89.0%) patients visited within 3 weeks. Nimodipine was prescribed to all patients on the day of visit. Accordingly, time from onset to treatment was the time from onset to visit in 81 (98.8%) patients and was longer in only one patient who did not take nimodipine during the first 3 weeks from the prescription. The median time from onset to treatment was 8 days (IQR 4–15), which nearly equals the time from onset to visit (median, 7 days; IQR, 4–15).

When grouped into tertiles, 26, 29, and 27 patients were classified into earliest (<6 days), early (6–13 days), and late (≥14 days) treatment groups, respectively. Patient demographics and characteristics according to treatment groups are described in Table 2. No significant difference was observed in terms of demographics and headache characteristics between treatment groups, although the mode of visit differed significantly (*p* < 0.001). BP surge was more frequently detected in the earliest treatment group (*p* = 0.026).

**Table 2:** Patient demographics and characteristics according to the timing of treatment.

	**Earliest (<6 days) (*n* = 26)**	**Early (6–13 days) (*n* = 29)**	**Late (≥14 days) (*n* = 27)**	***p***
Age (years)	51 (46–57)	53 (49–59)	51 (46–55)	0.321
Female sex	23 (88.5%)	24 (82.8%)	20 (74.1%)	0.428
Mode of recruitment				<0.001
ER	23 (88.5%)	17 (58.6%)	4 (14.8%)	<0.001
Outpatient	2 (7.7%)	12 (41.4%)	16 (59.3%)	0.001
Inpatient	1 (3.8%)	0 (0.0%)	7 (25.9%)	0.002
Headache severity (NRS)	9 (8–10)	10 (9–10)	9 (7–10)	0.105
Etiology				0.380
Postpartum	1 (3.8%)	1 (3.4%)	0 (0.0%)	
Drug	0 (0.0%)	0 (0.0%)	2 (7.4%)	
Idiopathic	25 (96.2%)	28 (96.6%)	25 (92.6%)	
Comorbid migraine	4 (15.4%)	5 (17.2%)	3 (11.1%)	0.795
Triggered by typical precipitants				0.448
At least one precipitant	19 (73.1%)	25 (86.2%)	20 (74.0%)	
Multiple precipitants	10 (38.5%)	9 (31.0%)	11 (40.7%)	
Typical precipitants				
Sexual activity	1 (3.8%)	1 (3.4%)	2 (7.4%)	0.837
Exertion	0 (0.0%)	0 (0.0%)	2 (7.4%)	0.204
Valsalva-like maneuver	10 (38.5%)	17 (58.6%)	8 (29.6%)	0.079
Emotion	8 (30.8%)	5 (17.2%)	4 (14.8%)	0.304
Bathing and/or showering	6 (23.1%)	5 (17.2%)	8 (29.6%)	0.547
Bending	4 (15.4%)	7 (24.1%)	7 (25.9%)	0.611
Focal neurological deficit	3 (11.5%)	3 (10.3%)	3 (11.1%)	>0.999
Neurological complication at the time of presentation				
Any	3 (11.5%)	2 (6.9%)	2 (7.4%)	0.789
Seizure	0 (0.0%)	1 (3.4%)	0 (0.0%)	>0.999
Ischemic stroke	0 (0.0%)	1 (3.4%)	0 (0.0%)	>0.999
Cortical SAH	1 (3.8%)	0 (0.0%)	2 (7.4%)	0.306
Intracerebral hemorrhage	1 (3.8%)	0 (0.0%)	0 (0.0%)	0.317
PRES	1 (3.8%)	0 (0.0%)	0 (0.0%)	0.317
BP surge	15 (57.7%)	10 (34.5%)	6 (22.2%)	0.026
Extent of vasoconstriction	4 (3–6)	5 (4–8)	5 (3–6)	0.908

*Data are presented as median (IQR) or number (percent). BP, blood pressure; ER, emergency room; NRS, numeric rating scale; PRES, posterior reversible encephalopathy syndrome; SAH, subarachnoid hemorrhage*.

### Clinical Course of RCVS

The median clinical course was 6 (IQR 2–10) days. The lengths of clinical courses varied, from a single attack (*n* = 15, 18.3%) to >30 days (*n* = 1, 1.2%). In 71 (86.6%) patients, TCHs remitted immediately after the start of nimodipine treatment. Fifty-five patients (67.1%) had a pretreatment remission period, among which 10 (12.2%) showed a prolonged remission suggestive of spontaneous remission. Among 11 patients (13.4%) who had recurrent TCHs despite treatment, the dose of nimodipine was increased in seven patients (8.5%). All but two had no recurrence immediately after the dose increment. One patient who had a large amount of intracerebral and subarachnoid hemorrhages received additional intravenous and intra-arterial administration of nimodipine for 1–2 weeks after onset because of recurrent transient ischemic attacks. In one patient, the nimodipine dose was escalated because of persistent headache, although TCHs had already remitted.

### Effect of Nimodipine on the Clinical Course of RCVS

Clinical outcomes were compared between treatment groups (Table 3). The clinical course was shortest in the earliest treatment group (median, 2 days; IQR, 0–4) and was longer in the early (median, 7 days; IQR, 3–9) and late (median, 10 days; IQR, 6–18) treatment groups (all subgroups after Bonferroni correction for multiple comparisons, *p* < 0.001; Table 3). In the earliest treatment group, most patients (*n* = 24, 92.3%) experienced remission in the first week. A trend was observed toward a higher proportion of single attacks in the earliest treatment group (34.6%; *p for trend* = 0.033). In the analysis of the total number of TCHs, 7 patients (earliest treatment group, *n* = 1; early treatment group, *n* = 2; and late treatment group, *n* = 4) were excluded due to inaccurate counting. The median total number of TCHs score was 2 (IQR 1–2), 4 (IQR 2–6) and 3 (IQR 2–5) in earliest, early and late treatment group, respectively. A trend was observed toward a lower frequency of TCHs in the earlier treatment group (*p for trend* = 0.031). TCHs remitted after nimodipine treatment in most patients regardless of the timing of treatment (88.5, 86.2, and 85.2% in the earliest, early, and late treatment groups, respectively; Table 3). Neurological complications at presentation were overall infrequent and did not differ among the treatment groups (*p* = 0.789; Table 2). After nimodipine administration, four patients (4.9%) had transient focal neurological deficits and only one patient (1.2%) developed cerebral infarction. No difference was observed in post-treatment complication rates among the groups (*p* = 0.317).

**Table 3:** Clinical outcomes according to the timing of treatment.

	**Earliest (<6 days) (*n* = 26)**	**Early (6–13 days) (*n* = 29)**	**Late (≥14 days) (*n* = 27)**	*p* for trend^*^
Clinical course (days)	2 (0–4)	7 (3–9)	10 (6–18)	<0.001
Ended in single attack	9 (34.6%)	3 (10.3%)	3 (11.1%)	0.033
Total number of TCHs^†^	2 (1–2)	4 (2–6)	3 (2–5)	0.031
Remission immediately after treatment	23 (88.5%)	25 (86.2%)	23 (85.2%)	0.843
Any neurological complication after treatment	1 (1.2%)	0 (0.0%)	0 (0.0%)	0.317
Neurological complications in total^‡^	3 (11.5%)	2 (6.9%)	2 (7.4%)	0.636

* *Linear-by-linear association analysis and the Jonckheere–Terpstra test were applied to categorical and continuous variables, respectively*.

† *Data from 7 patients (earliest treatment, n = 1; early treatment, n = 2; and late treatment, n = 4) were excluded due to inaccurate counting*.

‡ *Any neurological complications before and after treatment*.

### Survival Analysis for Clinical Course

**Figure 3:**
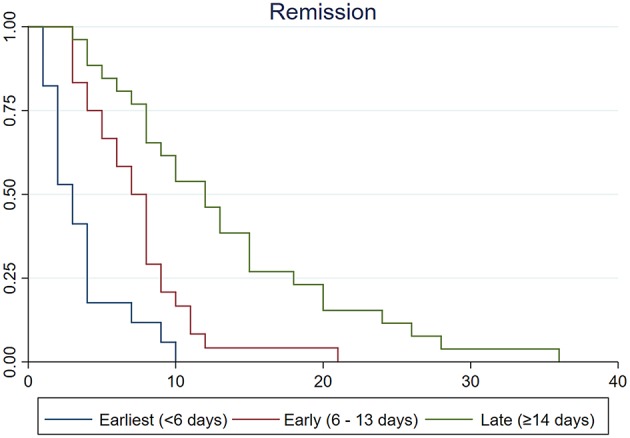
Kaplan–Meier survival curves among earliest (<6 days, blue line), early (6–13 days, red line), and late (≥14 days, green line) treatment groups. Vertical lines indicate remission of thunderclap headaches. Time from remission was clearly associated with the timing of treatment (log-rank test *p* < 0.001). The median time from remission was significantly shorter in earliest treatment group than in early (log-rank test *p* = 0.010) and late treatment groups (log-rank test *p* < 0.001).

The results of Kaplan–Meier analysis are summarized in Table 4 and Figure 3. Survival curves according to treatment groups showed that clinical courses clearly distinguished by the timing of treatment (all log-rank *p* < 0.05 after Bonferroni correction for multiple comparisons; Table 4 and Figure 3). Other variables such as age, sex, extent of vasoconstriction, neurological complications, and length of pretreatment remission period were not significantly associated with clinical course (Table 4). Although the Kaplan–Meier analysis yielded a possible association between PRES and clinical course, we did not regard it as significant because the PRES group comprised only one patient who had a very short clinical course (1 day).

**Table 4:** Predicted clinical course in patients with RCVS by predefined prognostic factors.

	**No. (%)**	**Clinical course, median (95% CI)**	p^*^	***p*^†^**
Treatment groups			<0.001	
Earliest (<6 days)	26 (31.7%)	2 (1–3)		Reference
Early (6–13 days)	29 (35.4%)	7 (4–10)		0.010
Late (≥14days)	27 (32.9%)	10 (5–15)		<0.001
Age			0.388	
<52 years	39 (47.6%)	6 (3–9)		
≥52 years	43 (52.4%)	5 (1–9)		
Sex			0.848	
Female	67 (81.7%)	5 (3–7)		
Male	15 (18.3%)	7 (5–9)		
Extent of vasoconstriction			0.928	
<5 segments	40 (48.8%)	4 (2–6)		
≥5 segments	42 (51.2%)	7 (5–9)		
Pretreatment remission period			0.427	
<2 days	37 (45.1%)	6 (3–9)		
≥2 days	45 (54.9%)	5 (2–8)		
Focal neurological deficit			0.802	
No	73 (89%)	5 (3–7)		
Yes	9 (11%)	8 (5–11)		
**Neurological complication at the time of presentation**				
Any complications			0.358	
No	75 (91.5%)	5 (2–10)		
Yes	7 (8.5%)	6 (0–9)		
Seizure			0.724	
No	81 (98.8%)	5 (3–7)		
Yes	1 (1.2%)	6		
Ischemic stroke			0.833	
No	81 (98.8%)	5 (3–7)		
Yes	1 (1.2%)	9		
Cortical SAH			0.239	
No	79 (96.3%)	6 (4–8)		
Yes	3 (3.7%)	1 (0–3)		
Intracerebral hemorrhage			0.716	
No	81 (98.8%)	5 (3–7)		
Yes	1 (1.2%)	10		
PRES			0.035	
No	81 (98.8%)	6 (4–8)		
Yes	1 (1.2%)	1		

*Unless otherwise specified, continuous variables were categorized using the median as a cutoff value*.

* by log-rank tests pooled over strata;

† *by the log-rank test compared with the earliest treatment group, with Bonferroni correction for multiple comparisons*.

*CI, confidence interval; PRES, posterior reversible encephalopathy syndrome; RCVS, reversible cerebrovascular constriction syndrome; SAH, subarachnoid hemorrhage*.

When a univariable Cox proportional hazards model was applied to possible predictors of the clinical course, time from onset to treatment was significantly associated with earlier remission (i.e., a shorter clinical course) (HR = 0.91 per 1-day delay in treatment, 95% CI = 0.879–0.948, *p* < 0.001; Table 5). Associations among age, sex, extent of vasoconstriction, pretreatment neurological complications, and length of pretreatment remission period were not significant. In the final multivariable model, the timing of treatment was independently associated with the length of clinical courses (adjusted HR = 0.75 per 1-day in delay in treatment; 95% CI, 0.693–0.802; *p* < 0.001). The extent of vasoconstriction was associated with a longer clinical course, but statistical significance was not reached (adjusted HR = 0.94 for each vasoconstricted segment; 95% CI, 0.874–1.006; *p* = 0.075).

**Table 5:** Univariable and multivariable Cox proportional hazards model results for the clinical course of RCVS.

	**Univariable**			**Multivariable**		
	**HR**	**95% CI**	***p*-value**	**HR**	**95% CI**	***p***
Age	1.00	0.98–1.02	0.760	1.00	0.97–1.02	0.789
Male sex	1.05	0.60–1.86	0.860	1.37	0.70–2.70	0.359
Extent of vasoconstriction	0.98	0.91–1.05	0.530	0.94	0.87–1.01	0.075
Time from onset to treatment (days)	0.91	0.88–0.95	<0.001	0.75	0.69–0.80	<0.001
Pretreatment remission period (days)	1.01	0.98–1.05	0.490	1.38	1.26–1.50	<0.001
Neurological complications before treatment	1.67	0.72–3.89	0.233	0.93	0.48–1.79	0.821

*CI, confidence interval; HR, hazard ratio*.

### Safety of Nimodipine

Eight patients (9.8%) reported mild side effects from nimodipine treatment. Side effects included general weakness (*n* = 3), dizziness (*n* = 3), sense of hunger (*n* = 1), paresthesia (*n* = 1), drowsiness (*n* = 1), abdominal discomfort (*n* = 1), tiredness (*n* = 1), and bilateral leg swelling (*n* = 1). All but one patient tolerated nimodipine treatment. Systemic blood pressure was followed-up in all patients at a median of 16 (IQR 10–30) days after treatment. Compared with baseline SBP (median, 137 mmHg; IQR, 123.5–153.0) and DBP (median, 84.0 mmHg; IQR, 74.5–93.5), post-treatment BPs were slightly reduced but remained within the normal range (median, 125 mmHg [IQR 113–136] and 75 mmHg [IQR 71–84] for SBP and DBP, respectively).

## Discussion

In this prospective observational study, we found that the clinical course of RCVS differed according to the timing of nimodipine treatment. Earlier treatment was independently associated with a shorter clinical course. Nimodipine effectively prevented recurrent TCHs in most patients. When treated within the first week, patients were more likely to have only a single TCH during the entire clinical course.

The role of nimodipine in the treatment of RCVS has not been validated in previous studies. Although the strong efficacy of nimodipine in preventing recurrent TCHs was suggested in a headache clinic-based cohort (2), this result was challenged by other studies where no significant association between the use of nimodipine and excellent neurological outcomes was found (4, 7). Because no consensus guidelines have been available for the treatment of RCVS, different strategies have been used in different clinical settings (7). In such situations, the selection of treatment modality might have been influenced by neurological status; thus, unbiased estimation of the effects of nimodipine based on such retrospective studies is difficult. We were able to overcome such limitations in an observational study because nimodipine is considered standard therapy for RCVS in our hospital. All patients were given nimodipine immediately after other causes of TCH were excluded, regardless of neuroimaging results. This enabled relatively unbiased estimation of the effect of nimodipine according to the timing of treatment.

In previous studies, nimodipine was not associated with reduced complication rates (4, 7). In our study, the proportion of neurological complications did not differ according to the treatment group, but complications rarely developed after treatment. In our previous research (19), the complication rate of RCVS in our cohort (15.4%) was comparable to that in a Taiwanese cohort (11.7%) but much lower than in other cohorts (28–81%) (4–7). We discussed the reasons for discrepancies in our previous studies (17, 19): different ethnicities, national rates of illicit drug usage, and differences in study setting and healthcare system. In the present study, the complication rate in our institute decreased to 8.5%, which might be attributable to an improved awareness of RCVS in our hospital leading to a rapid referral of patients to us, early diagnosis of RCVS, immediate start of nimodipine, and identification of milder cases that could have previously been underdiagnosed. Under such circumstances, our data might not be suitable to test the effect of nimodipine on the prevention of neurological complications.

In this study, we investigated the effect of nimodipine on the recurrence of TCHs in patients with RCVS. Our observation highlights that the clinical course of RCVS can be modulated by earlier treatment. Recurrent TCHs within the first 2–3 weeks are the most characteristic presentation of RCVS (3–5, 7, 20). However, the clinical course was considerably shortened by earlier treatment in our study. Lower frequency of TCHs were also observed in the earlier treatment group. Furthermore, more than one-third of patients had only a single TCH when treated within the first week after onset. This proportion was higher than expected because only a minority of patients with RCVS had a single TCH in the literature (4, 6, 7). Although some of our patients showed a possibility of spontaneous remission before treatment, the association between earlier treatment and shorter clinical course remained significant when adjusted for the pretreatment remission period. Our findings suggest that nimodipine can exert a pathophysiological effect on RCVS. Although the pathophysiology of RCVS remains unclear, TCHs are postulated to be caused by dilatation of distal small arteries (8, 9, 21). From this perspective, we hypothesize, based on our results, that the early use of nimodipine may stabilize vasoconstrictions and subsequent vasodilatations of distal small arteries, which might be beneficial to further preventing centripetal propagation of vasoconstrictions (4, 22). This hypothesis should be tested in a prospective, longitudinal study with serial angiographies in patients with RCVS.

From a safety perspective, no serious complication such as symptomatic hypotension was observed in patients treated with nimodipine. This might have been due to selective effects of nimodipine on cerebral vessels (2, 4). Systemic nimodipine may precipitate steal phenomena in narrowed arteries. However, almost none our study patients developed ischemic complications while receiving nimodipine, although this should be carefully interpreted in consideration of our study setting (more than one-third of patients from an outpatient headache clinic), population (patients who visited the hospital because of TCH), and the extremely low complication rate.

The prospective setting and structured follow-up of the clinical course are the major strengths of our study. In addition, the large number of patients is another strength. In our hospital, more than 2,500 first-visit patients per year visit the headache clinic. This was enabled by the Korean national health insurance system covering almost 97% of the population and allowing an easy access to tertiary hospital. However, our study is not without limitations. First, our study did not include placebo controls. However, we considered that a placebo-controlled trial might be unethical because patients with RCVS are at risk of neurological complications and several previous studies demonstrated a beneficial effect of nimodipine. Despite the lack of a placebo control, our study results may be less biased because the diagnosis and treatment of TCH is based on a clinical protocol in our hospital. Second, as discussed in our recent studies, patients in our cohort had fewer secondary causes and lower complication rates compared to other cohorts (4, 6, 7). Although we consider this a consequence of earlier identification of RCVS in our clinical environment rather than a limitation, our study results might not be generalizable to different clinical settings in which the proportion of secondary causes (e.g., postpartum or illicit drug usage) is high and neurological complications are frequent. Third, baseline characteristics slightly differed between treatment groups, which suggests that patients who visited ER have more chance to get earlier treatment. Although the disease severity of RCVS did not differ, thus our results would not be significantly biased, some unmeasured confounders may exist. Fourth, we did not use the total number of TCHs as outcome variables because several patients could not exactly recall it particularly when they had numerous attacks. We took the disease duration, i.e., time from onset to remission, as an outcome variable, which might be less robust to account for disease severity. Fifth, the dose of nimodipine in our clinical practice is slightly less than the dose specified in other studies (5, 6). To date, no guidelines specified the recommended dose of nimodipine for RCVS. In literatures, authors have recommended various regimens such as oral nimodipine 30–60 mg every 4–8 h based on effectiveness of pain relief and clinical severity (23), oral nimodipine 60 mg every 4–8 h (24), oral nimodipine 30 to 60 mg every 4 h (5, 6), or intravenous nimodipine 0.5–2 mg/h (5, 6). In our study, initial treatment was started at a dose of 30 mg every 8–12 h per day (median, 1.5 mg/kg/day). We started with the low-dose nimodipine because a systemic administration of high-dose nimodipine can lead to vascular steal phenomenon. Higher dose was used when TCH recurs despite of the treatment or patients had neurological complications. We have reported good outcome and low rate of neurological complications of our low-dose strategy in our headache-clinic based study (19). In this study, we showed the efficacy and safety of low dose nimodipine in the treatment of RCVS. However, this might not be generalized because patient characteristics and clinical setting are different between countries. Sixth, the clinical course might be affected by natural disease course of RCVS. It is known that RCVS is usually self-limited within 3–6 months, and TCH typically recur over a period of 1 to 4 weeks and then remit spontaneously. If the natural course is the only determinant of the duration (time from onset to remission) of recurrent TCHs, the clinical course may be similar regardless of the timing of nimodipine treatment. However, in our study, patients showed variable lengths of clinical courses which was mostly determined by the time of treatment.

In conclusion, we suggest the role of nimodipine in the treatment of RCVS. The clinical course of RCVS was shortened by earlier nimodipine treatment. In addition to preventing TCHs, beneficial effects of earlier nimodipine treatment on the progression of vasoconstriction and development of neurological complications should be investigated in future studies.

## Data Availability

Any data not published within this article will be shared, in an anonymized data, will be shared by request from any qualified investigator.

## Ethics Statement

The study received ethical approval from The Samsung Medical Center Institutional Review Board. Written consent was obtained for all patients at the inclusion visit.

## Author Contributions

SC: study design, analysis, interpretation of data, and drafting the manuscript. ML: study concept and design, acquisition of data, analysis of data, revising the manuscript for intellectual content, and drafting the manuscript. C-SC: study concept and critical revision of manuscript for intellectual content.

### Conflict of Interest Statement

The authors declare that the research was conducted in the absence of any commercial or financial relationships that could be construed as a potential conflict of interest.
